# Diversity of Filamentous Fungi Isolated From Some Amylase and Alcohol-Producing Starters of India

**DOI:** 10.3389/fmicb.2020.00905

**Published:** 2020-05-29

**Authors:** Anu Anupma, Jyoti Prakash Tamang

**Affiliations:** Department of Microbiology, DAICENTRE (Department of Biotechnology-National Institute of Advance Industrial Science and Technology (DBT-AIST) International Centre for Translational and Environmental Research) and Bioinformatics Centre, School of Life Sciences, Sikkim University, Gangtok, India

**Keywords:** filamentous molds, amylolytic starter, India, *Mucor*, *Rhizopus*, *Aspergillus*, *Penicillium*

## Abstract

Filamentous fungi are important organisms in traditionally prepared amylase and alcohol-producing dry starters in India. We collected 40 diverse types of amylase and alcohol-producing starters from eight states in North East India viz. *marcha*, *thiat*, *humao*, *hamei*, *chowan*, *phut*, *dawdim*, and *khekhrii*. The average fungal population was 4.9 × 10^5^ cfu/g with an average of pH 5.3 and 10.7%, respectively. In the present study, 131 fungal isolates were isolated and characterized based on macroscopic and microscopic characteristics and were grouped into 44 representative fungal strains. Based on results of morphological characteristics and ITS gene sequencing, 44 fungal strains were grouped into three phyla represented by Ascomycota (48%), Mucoromycota (38%), and Basidiomycota (14%). Taxonomical keys to species level was illustrated on the basis of morphological characteristics and ITS gene sequencing, aligned to the fungal database of NCBI GenBank, which showed seven genera with 16 species represented by *Mucor circinelloides* (20%), *Aspergillus sydowii* (11%), *Penicillium chrysogenum* (11%), *Bjerkandera adusta* (11%), *Penicillium citrinum* (7%), *Rhizopus oryzae* (7%), *Aspergillus niger* (5%), *Aspergillus flavus* (5%), *Mucor indicus* (5%) *Rhizopus microsporus* (5%), *Rhizopus delemar* (2%), *Aspergillus versicolor* (2%), *Penicillium oxalicum* (2%), *Penicillium polonicum* (2%), *Trametes hirsuta* (2%), and *Cladosporium parahalotolerans* (2%). The highest Shannon diversity index *H* was recorded in *marcha* of Sikkim (*H*: 1.74) and the lowest in *hamei* of Manipur (*H*: 0.69). Fungal species present in these amylolytic starters are morphologically, ecologically and phylogenetically diverse and showed high diversity within the community.

## Introduction

Drinking alcoholic beverages has a cultural connotation in India from the Indus Valley Civilization dating back to 8,000 years (Sarkar et al., 2016), mostly through fermentation (Singh et al., 2010) and distillation (Achaya, 1991). Traditionally malting, brewing (such as beer), and vinification (fermentation of grapes into wine) processes are unknown in Indian food culture. Instead, traditional alcoholic beverages are prepared either by natural fermentation of plants or cereals, or by using traditionally prepared dry starters in India (Tamang, 2010). Some ethnic people in India traditionally prepare amylase and alcohol-producing starters to ferment alcoholic beverages for home consumption, which are known by different names in different languages spoken locally in regions such as *marcha* in Sikkim and Darjeeling hills, *thiat* in Meghalaya, *humao* in Assam, *hamei* in Manipur, *chowan* in Tripura, *phut* in Arunachal Pradesh, *dawdim* in Mizoramand *khekhrii* in Nagaland (Anupma et al., 2018), *dhehli, balam, maler, treh*, and *bakhar* of Himachal Pradesh and Uttarakhand (Thakur et al., 2015), and *ranu dabai/goti* of West Bengal, Odisha and Jharkhand (Ghosh et al., 2015). Traditional methods of the preparation of Indian starters are almost the same with some differences in use of starch-rich substrates such as rice or wheat or barley, and wrapping materials either in fern fronds or dry paddy-straw, or in fresh leaves of locally available wild plants (Shrivastava et al., 2012; Tamang et al., 2016). Soaked, dewatered, and ground cereal (rice/wheat/barley) flours are mixed with some wild plants, with a few spices such as sun-dried chilies or garlics and supplemented with 1–2% of previously prepared dry starters in powder forms (“back-slopping method” for sub-culturing the microbiota) to make thick doughs with addition of water. Thoroughly mixed dough mixtures are made into round or flat cakes of varying shapes and sizes, placed on fresh ferns or other plant leaves/dry paddy straws and allowed to ferment under semi-anaerobic conditions for 2–3 days at room temperature inside the room. After desirable fermentation, fermented doughs are then sun dried for 2–3 days to obtain dry starters which are exclusively used to ferment cereals into mild/strong alcoholic beverages (Tamang, 2010; Anupma et al., 2018). However, *khekhrii*, a dry starter from Nagaland in India is prepared by naturally fermenting sprouted-rice grains which are then dried in the sun to obtain dry starter granules to prepare an alcoholic beverage locally called *zutho*. Indian amylase and alcohol-producing starters are similar to starters from Asian countries such as *daqu* or *chiu* from China (Zheng et al., 2012), *benh* from Vietnam (Dung et al., 2007), *nuruk* from Korea (Jung et al., 2012), *ragi* from Indonesia (Roslan et al., 2018), *bubod* from the Philippines (Fronteras and Bullo, 2017), *loogpang* from Thailand (Daroonpunt et al., 2016) and *dombea* or *medombae* from Cambodia (Ly et al., 2018).

Several species of filamentous molds (Hesseltine et al., 1988; Yang et al., 2011; Lv et al., 2012a; Chen et al., 2014; Das et al., 2017); yeasts (Hesseltine and Kurtzman, 1990; Tamang and Sarkar, 1995; Tsuyoshi et al., 2005; Jeyaram et al., 2008, 2011; Thanh et al., 2008; Fronteras and Bullo, 2017; Sha et al., 2017, 2018, 2019), and bacteria (Hesseltine and Ray, 1988; Tamang et al., 2007; Sha et al., 2017; Roslan et al., 2018) are found to coexist in traditionally prepared dry starters as “micro-resources” which have been sub-cultured to preserve essential microbiota for alcohol production by Asian people for centuries (Tamang et al., 2020). Filamentous fungi present in traditional starters from Asia have several functionalities such as saccharification (Lee and Lee, 2002; Thapa and Tamang, 2004), liquefaction (Suesse et al., 2016), and ethanol production (Dung et al., 2007; Chen et al., 2014) to produce different types of low-alcoholic beverages and high-alcoholic distilled liquor. Filamentous molds are also responsible for the quality of alcoholic beverages including nutritional values and organoleptic properties such as flavor, taste, and color (Zhang et al., 2015; Tamang et al., 2016). Taxonomical identification of filamentous molds isolated from traditionally prepared dry starters from India have not been reported yet except from *marcha* (Tamang et al., 1988; Sha et al., 2017, 2019), *thiat* (Sha et al., 2017, 2019), *amou*, and *perok-kushi* (Das et al., 2017). *Mucor circinelloides*, *Rhizopus chinensis*, and *Rhizopus stolonifer* were reported earlier from *marcha* samples collected from Nepal, Darjeeling, and Sikkim (Tamang et al., 1988; Tamang and Sarkar, 1995; Thapa and Tamang, 2006; Sha et al., 2017, 2018), *Amylomyces rouxii* and *Rhizopus oryzae* from samples of *amou*, and *perok-kushi*, traditional starters of Assam (Das et al., 2017). Sha et al. (2017) reported fungal Phylum Ascomycota (98.6%) followed by Mucoromycota (1.4%), while in *marcha* samples only Phylum Ascomycota by high-through sequencing was reported. The present study aimed to identify the filamentous molds isolated from eight different types of traditionally prepared starters from North East India, viz. *marcha, thiat, humao, hamei, chowan, phut, dawdim*, and *khekhriii*, to species level by morphological and molecular identifications, and to profile their diversity within the fungal community.

## Materials and Methods

### Sample Collection

**FIGURE 1:**
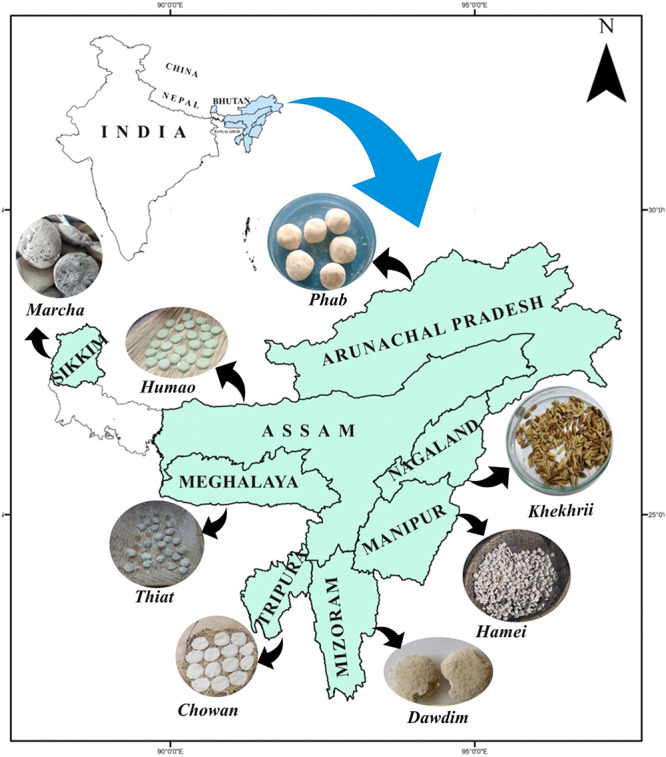
Location map of North East India showing collection sites of traditionally prepared dry starters.

A total of 40 samples of traditionally prepared dry starters viz *marcha* from Sikkim, *thiat* from Meghalaya, *humao* from Assam, *hamei* from Manipur, *chowan* from Tripura, *phut* from Arunachal Pradesh, *dawdim* from Mizoram, and *khekhrii* from Nagaland (Table 1) were collected directly from local markets and the homes of local producers in North East India (Figure 1) in pre-sterile containers. Dry starter samples were transported to the laboratory and stored in desiccators at room temperature as traditionally prepared dry starters have a shelf life of more than 1 year (Sha et al., 2018).

**TABLE 1:** Geographical locations, pH, moisture content, and fungal populations of dry starters from North East India.

**Sample (n^a^)**	**Region**	**Collection Site**	**Altitude (Meter)**	**Moisture content (%)**	**pH**	**cfu/g (×10^5^)**
*Marcha* (*n* = 8)	Sikkim	Gangtok	1637	11.6(10.1−12.1)	5.2(4.9−5.7)	5.0(4.8−5.1)
		Basilakha	906			
		Pakyong	1341			
		Recabe	1072			
*Thiat* (*n* = 4)	Meghalaya	Shillong	1550	9.4(8.7−10.0)	4.7(4.5−5.0)	4.8(4.5−5.1)
		Non-grem	1547			
*Humao* (*n* = 7)	Assam	Kokrajhar	49	9.7(8.8−10.6)	4.9(4.6−5.2)	4.6(4.3−5.3)
		Jorhat	95			
		Sivsagar	93			
		Moran	100			
*Hamei* (*n* = 3)	Manipur	Kangchup	773	8.5(8.0−9.6)	4.6(4.1−5.4)	2.6(2.5−3.2)
		Kakching	769			
		Phayeng	813			
*Chowan* (*n* = 4)	Tripura	Bangsul	116	9.1(9.0−9.3)	5.6(5.4−5.9)	3.1(3.0−3.4)
		Dharmanagar	98			
*Phut* (*n* = 6)	Arunachal Pradesh	Doimukh	152	11.2(11.4−11.8)	5.4(5.5−5.7)	5.6(4.9−5.9)
		Pasighat	155			
		Itanagar	361			
		Banderdewa	462			
		Nirjuli	151			
*Dawdim* (*n* = 3)	Mizoram	Saitual	438	13.7(13.1−13.9)	6.2(6.1−6.3)	7.4(7.1−7.9)
*Khekhrii* (*n* = 5)	Nagaland	Kohima	1092	12.8(12.3−13.1)	5.6(5.5−5.9)	6.0(5.7−6.8)

**^*a*^n = number of samples.**

### Analysis of pH and Moisture Content

The pH of homogenized samples was recorded by digital pH-meter (Orion 910003, Thermo Fisher Scientific, United States). The moisture content of the samples was estimated by a moisture analyzer (OHAUS/MB-45, United States).

### Microbiological Analysis

Each dry sample starter was taken from the desiccator, then crushed coarsely by sterile spatula and 10 g of the crushed powered sample was homogenized with 90 mL of 0.85% physiological saline in a stomacher lab blender 40 (Seward, United Kingdom) for 2 min to obtain serial dilutions. One milliliter of each diluted sample (10^–4^, 10^–5^, 10^–6^, and 10^–7^) was poured onto malt extract agar (M137, HiMedia, Mumbai, India) and potato dextrose agar (M096, HiMedia, Mumbai, India) with an addition of antibiotics (1% streptomycin) to suppress the growth of bacteria, and plates were then incubated under 28°C and observed for the appearance of colonies for up to 1 week. The colonies that appeared on plates were counted as a colony forming unit (cfu/g) on the dry weight of starters. Colonies were selected on the basis of macroscopic and microscopic characteristics. Selected filamentous molds were sub-cultured on new plates and purified and stored on slants at 4°C for further studies.

### Morphological and Physiological Identification

For each isolate, one- or three-point inoculations on petri plates containing ~25 mL of media were applied. Fungal morphology was studied macroscopically by observing the colony features (surface color, reverse side color, shape, and diameter), and microscopically by observation of fruiting bodies using a stereomicroscope, and the vegetative and asexual stages were observed by a DE/Axio Imager A1 microscope (Carl Zeiss, Oberkochen, Germany) after staining freshly grown mycelia stained with cotton blue in MEA plates (Gaddeyya et al., 2012). Filamentous molds were identified on the basis of morphological features using the taxonomical keys described by Samson et al. (2004) and Pitt and Hocking (2009).

### Genomic DNA Extraction

The genomic DNA was extracted from mold cultures following the methods of Umesha et al. (2016). Mycelial mass from the culture plate was scraped out by a sterile surgical blade and ground in a sterile mortar and pestle using 500 μL extraction buffer [100 mM Tris–HCl (pH 8.0), 20 mM EDTA (pH 8.0), 1.4 M NaCl, 2% CTAB, and 0.2% 2 mercaptoethanol]. The mixture was transferred to a fresh 1.5 mL tube with addition of 4-μL RNase, vortexed and incubated for 60 min at 37°C, and kept in a water bath for 60 min at 55°C. 500 μL phenol: chloroform: isoamyl alcohol (25:24:1) was added to the solution, mixed thoroughly for 5 min, and then centrifuged at 14,000 rpm for 10 min. The aqueous clear phase was recovered and mixed with chloroform: isoamyl alcohol (24:1), centrifuged at 12,000 rpm for 5 min, and the aqueous phase was recovered, adding 0.8 volume of cold 7.5 M ammonium acetate and 0.54 volume of ice-cold isopropanol, and finally mixed well and stored overnight for precipitation of DNA in a deep freezer. The solution was centrifuged at 14,000 rpm for 3 min and precipitated with absolute ethanol to recover DNA. The DNA was then rinsed twice with 1 mL of 70% ethanol and resuspended in 100 μL of 1X TE [200 mM Tris–HCl (pH 8.0), 20 mM EDTA (pH 8.0)] buffer for further use and stored at −20°C. The quality of DNA was checked on agarose gel and the concentration was measured using a nanodrop spectrometer (ND-1000 spectrometer, NanoDrop Technologies, Willington, United States) (Kumbhare et al., 2015).

### PCR Amplification

Polymerase chain reactions (PCR) of the internal transcribed spacer (ITS) region of filamentous molds was amplified using the primer ITS1 (5′-TCCGTAGGTGAACCTGCGG-3′) and ITS4 (5′-TCCTCCGCTTATTGATATGC-3′) (Adekoya et al., 2017). PCR reactions were performed in 25 μL of PCR pre-master mix solution (Promega, United States). The amplification steps were followed: initial denaturation at 94°C for 5 min followed by 35 cycles consisting of 94°C for 1 min, 54°C for 1 min, and 72°C for 1 min, respectively; and a final extension at 72°C for 10 min in a Thermal Cycler (Applied biosystems-2720, United States). The PCR products were verified by electrophoresis on 1.0% agarose gel containing 0.7 mg/mL of ethidium bromide and visualized under UV light (Gel doc 1000, Bio-Rad, 97-0186-02, United States). Approximate size of amplicons was determined using standard molecular markers (Himedia-100 bp DNA ladder, Mumbai, India).

### Purification of the PCR Amplicons

The amplified PCR products were purified using PEG (polyethylene glycol)-NaCl (sodium chloride) and precipitation solution (20% w/v of PEG, 2.5 M NaCl) with the addition of 0.6 volumes of 20% PEG-NaCl to the final volume of the PCR products (Schmitz and Riesner, 2006). The mixture was centrifuged at 12,000 rpm for 30 min, incubated at 37°C for 30 min, the aqueous solution was discarded, and the pellet was washed twice with 1 mL ice cold 70% freshly prepared ethanol (70%). The collected pellet was then air dried prior to elution in 20 μL of nuclease-free water, and finally, the purified product was loaded in 1% agarose gel.

### ITS Sequencing

PCR-amplified products had been sequenced in a forward and reverse direction using ITS1 primer (5′-TCCGTAGGTGAACCTGCGG-3′) and ITS4 primer (5′-TCCTCCGCTTATTGATATGC-3′), respectively, as per the method described by Martin and Rygiewicz (2005). The PCR reaction was carried out in 50 μL reaction volume containing 2.0 mM MgCl2, 0.2 μM each primer, 0.2 mM dNTP, 0.5 mg [mL]^–1^ bovine serum albumin (BSA) and 0.04 U [μL]^–1^ tTaq DNA polymerase on a thermal cycler equipped with a heated lid. The thermal program included initial denaturation, enzyme activation at 95°C (6–10 min) followed by 35 cycles to complete the step [95°C (1 min), 40°C (2 min) and 72°C (1 min)] and one cycle at 72°C (10 min). The amplified products were sequenced by an automated DNA Analyzer (ABI 3730XL Capillary Sequencers, Applied Biosystems, Foster City, CA, United States). These high-quality, double-stranded sequence data were analyzed with the help of the BLASTn program and multiple sequence alignment.

### Bioinformatics

The qualities of the raw sequences were checked by Sequence Scanner version 1.0 (Applied Biosystems, Foster City, CA, United States) and were edited using software ChromasPro version 1.34. Sequences were compared with sequence entries in the GenBank of NCBI (National Center for Biotechnology Information)^1^ using the Basic Local Alignment Search Tool for nucleotides (BLASTn) on the NCBI website (Pinto et al., 2012). For phylogenetic analysis, the available sequence of similar related organisms was retrieved in FASTA format and aligned using the clustal-W. Sequence alignment and a phylogenetic tree were constructed using MEGA7.0 software by Neighbor-Joining methods using 1000-bootstrap replicates (Lutzoni et al., 2004).

### Statistical Analysis

Percentages of frequency and relative density of fungal species in samples were determined as per the method described by Doi et al. (2018). Frequency (%) was calculated by the equation:

$$\text{Frequency}\left(\%\right)=\frac{\mathrm{Number}\,\mathrm{of}\,\mathrm{quadrats}\,\mathrm{in}\,\mathrm{which}\,\mathrm{the}\,\mathrm{species}\,\mathrm{occurred}}{\mathrm{Total}\,\mathrm{number}\,\mathrm{of}\,\mathrm{quadrats}\,\mathrm{studied}}\times 100$$

Relative Density (%) was calculated by the equation:

$$\text{Density}=\frac{\mathrm{Total}\,\mathrm{number}\,\mathrm{of}\,\mathrm{individuals}\,\mathrm{of}\,a\,\mathrm{species}\,\mathrm{in}\,\mathrm{all}\,\mathrm{quadrats}}{\mathrm{Total}\,\mathrm{number}\,\mathrm{of}\,\mathrm{quadrats}\,\mathrm{studied}}\times 100$$

Diversity indexes of filamentous molds in samples were calculated by species richness (R), Shannon’s diversity index (H), and species evenness (E) (Panda et al., 2010) using PAST (Paleontological STatistics) software version 3.26 (Hammer et al., 2001).

### Nucleotide Sequence Accession Numbers

The sequences obtained in this study were deposited at the GenBank-NCBI database under accession numbers: MK396469–MK396484, MK396486–MK396500, MK778442–MK778449, and MK796041–MK796045.

## Results

### Microbial Load, pH, and Moisture

The microbial load of filamentous molds in 40 samples of traditionally prepared dry starters collected from different regions of North East India were 2.5 to 7.9 × 10^5^ cfu/g (Table 1). The pH and moisture contents of all samples analyzed were pH 4.1–6.3 and 8.0–13.9%, respectively (Table 1).

### Morphological Characterization

We isolated 131 total fungal isolates from 40 different samples of traditionally prepared dry starters (*marcha*, *thiat*, *humao*, *hamei*, *chowan*, *phut*, *dawdim*, and *khekhrii*) collected from eight states of North East India (Table 1). Based on the morphological characteristics (such as color, texture, size, and appearance of colony), microscopic characteristics (sporangia, sporangiospores, chlamydospores, conidia, conidiophore, and rhizoid structure), 44 representative fungal isolates were grouped (seven isolates from *marcha*, five from *thiat*, six from *humao*, two from *hamei*, five from *chowan*, six from *phut*, six from *dawdim*, and seven from *khekhrii*). *Mucor*, *Rhizopus*, *Aspergillus, Penicillium*, and *Cladosporium* and a few unidentified basidiomycetes fungi were tentatively identified on the basis of detailed morphological characters using the taxonomical keys described by Samson et al. (2004) and Pitt and Hocking (2009) (Supplementary Table S1).

### Molecular Identification of Fungal Isolates

**FIGURE 2:**
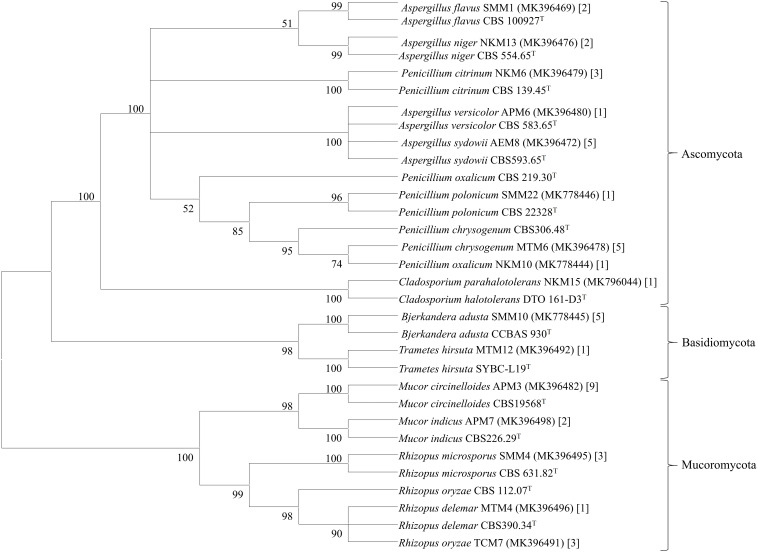
Molecular phylogenetic analysis of 44 filamentous fungal isolates from starters from North East India using the Neighbor-Joining method in MEGA7 software. The percentage of replicate trees in which the associated taxa clustered together in the bootstrap test (1000 replicates) is shown next to the branch. The tree is drawn to scale with branch lengths in the same units as those of the evolutionary distances used to infer the phylogenetic tree. The evolutionary distances were computed using the Kimura 2-parameter method and are in the units of the number of base substitutions per site. The phylogenetic tree branches are collapsed at 50%.

**FIGURE 3:**
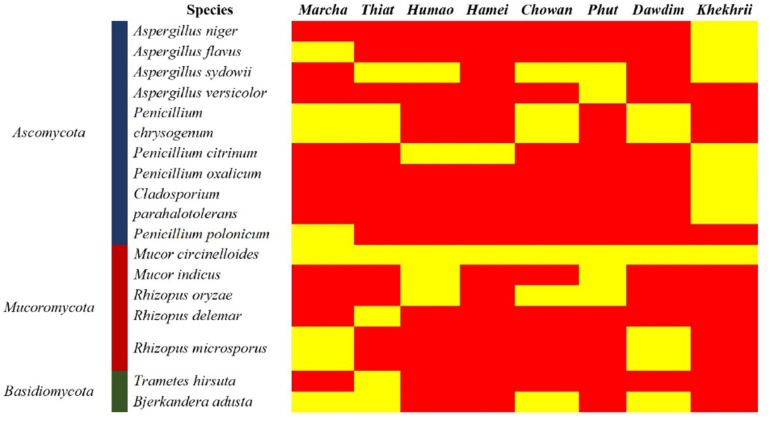
Heatmap showing the consensus species diversity resulted by ITS-region gene sequencing of filamentous fungal isolates. We used presence/absence value for fungal species to generate the heatmap, where the yellow color indicates the presence and red indicates absence.

Genomic DNA of each isolate of 44 representative fungal strains was extracted and PCR products were prepared for identification by ITS gene sequencing. DNA sequences of fungal isolates were assigned by comparison with those available in the GenBank of NCBI database using the ITS gene sequence (ITS1 and ITS4) based on the Basic Local Alignment Search Tool (BLAST) 2.0 program (Raja et al., 2017). The phylogenetic trees of nucleotide sequences of the 44 fungal isolates from the samples were constructed using the Neighbor-joining method with 1000 replicates bootstrap values (Figure 2). ITS gene sequencing results showed three fungal phyla represented by Ascomycota (48%), Mucoromycota (38%), and Basidiomycota (14%) (Figure 3). Distribution percentage of the phyla in the starter showed the highest percentage of Ascomycota (86%) in *khekhrii*, Mucoromycota (60%) in *dawdim*, and Basidiomycota (20%) in *chowan, dawdim*, and *thiat*, respectively. Phyla Ascomycota and Mucoromycota were present in all starters, whereas Basidiomycota was present only in *marcha*, *thiat, chowan*, and *dawdim.*

**FIGURE 4:**
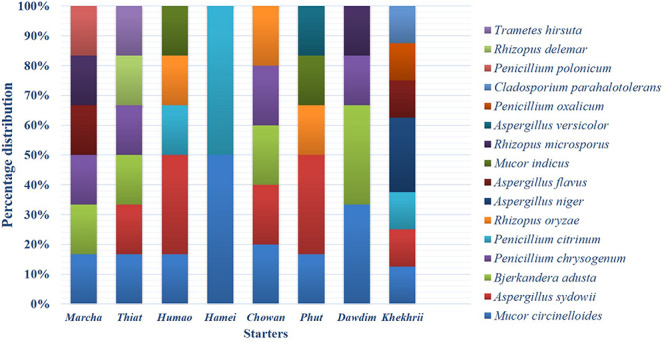
Abundance distribution of the filamentous fungi isolated from dry starters from North East India.

**FIGURE 5:**
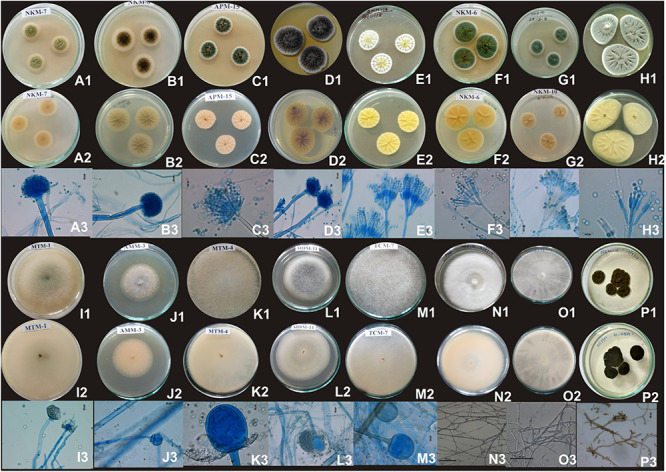
Images of colony morphology and microscopic features of filamentous molds that grew on MEA media: *Aspergillus flavus* colonies top **(A1)**, reverse **(A2)**, Conidiophores **(A3)**; *Aspergillus niger* colonies top **(B1)**, reverse **(B2)**, mature conidia globose conidial head contain conidia **(B3)**; *Aspergillus sydowii* colonies top **(C1)**, reverse **(C2)**, mature conidiophore with vesicle bearing conidiogenous metulae and phailides (biserate) **(C3)**; *Aspergillus versicolor* colonies top **(D1)**, reverse **(D2)**, conidial heads supported vesicles with which are biseriate with metulae about the same size of philiades **(D3)**; *Penicillium chrysogenum* colonies top **(E1)**, reverse **(E2)**, smooth-walled conidiophores stipes (150–280 μm) and biverticillate **(E3)**; *Penicillium citrinum* colonies top **(F1)**, reverse **(F2)**, conidiophores stipes (150–280 μm) and biverticillate, phialides ampuliform (flask-shaped) **(F3)**; *Penicillium oxalicum* colonies top **(G1)**, reverse **(G2)**, mature conidiophores monoverticillate, or biverticillate and asymmetrical, phialides were cylindrical; *Penicillium polonicum* colonies top **(H1)**, reverse **(H2)**, conidiophore were terverticillate, phialides **(H)**; *Mucor circinelloides* colonies top **(I1)**, reverse **(I2)**, mature sporangiosphores contain sporangiospores **(I3)**; *Mucor indicus* colonies top **(J1)**, reverse **(J2)**, mature sporangiosphores contain sporangiospores **(J3)**; *Rhizopus delemar* colonies top **(K1)**, reverse **(K2)**, globose sporangium **(K3)**; *Rhizopus oryzae* colonies top **(L1)**, reverse **(L2)**, sporangiophores were usually straight, mostly 10∼20 μm **(L3)**; *Rhizopus microsporus* colonies top **(M1)**, reverse **(M2)**, sporangia globose, smooth and released spore **(M3)**; *Trametes hirsuta* colonies top **(N1)**, reverse **(N2)**, hyphal structure **(N3)**; *Bjerkandera adusta* colonies top **(O1)**, reverse **(O2)**, dichotomously branched hyphae **(O3)**; *Cladosporium parahalotolerans* colonies top **(P1)**, reverse **(P2)**, conidiophores and conidial chain **(P3)**.

Based on results of morphological characteristics and ITS gene sequencing, 44 representative strains of filamentous molds isolated from traditionally prepared dry starters from India were grouped into seven genera with 16 species, which were represented by *Mucor circinelloides* (20%), *Aspergillus sydowii* (11%), *Penicillium chrysogenum* (11%), *Bjerkandera adusta* (11%), *Penicillium citrinum* (7%), *Rhizopus oryzae* (7%), *Aspergillus niger* (5%), *Aspergillus flavus* (5%), *Mucor indicus* (5%) *Rhizopus microsporus* (5%), *Rhizopus delemera* (2%), *Aspergillus versicolor* (2%), *Penicillium oxalicum* (2%), *Penicillium polonicum* (2%), *Trametes hirsuta* (2%), and *Cladosporium parahalotolerans* (2%) (Table 2 and Figure 4). Interestingly we detected few basidiomycetes fungi represented by *Bjerkandera adusta* and *Trametes hirsuta* in *marcha, thiat, chowan* and *dawdim* samples. Colony morphology and microscopic images of 16 species of seven genera of filamentous molds isolated from dry starters from India were illustrated for fungal taxonomy (Figure 5).

**TABLE 2:** Molecular identification of filamentous molds isolated from starters from North East India by ITS gene sequence (ITS1 and ITS4) based on BLAST.

**Product**	**Isolate code**	**Identity**	**GenBank accession number**	**Size in base pair (arbitrary primers)**
*Marcha*	SMM-1	*Aspergillus flavus*	MK396469	519
	SMM-3	*Mucor circinelloides*	MK396489	642
	SMM-4	*Rhizopus microsporus*	MK396495	703
	SMM-10	*Bjerkandera adusta*	MK778445	675
	SMM-16	*Penicillium chrysogenum*	MK396477	577
	SMM-22	*Penicillium polonicum*	MK778446	582
	SMM-35	*Penicillium chrysogenum*	MK778447	552
*Thiat*	MTM-1	*Mucor circinelloides*	MK396487	636
	MTM-4	*Rhizopus delemar*	MK396496	768
	MTM-6	*Penicillium chrysogenum*	MK396478	583
	MTM-12	*Trametes hirsuta*	MK396492	637
	MTM-16	*Bjerkandera adusta*	MK396500	651
*Humao*	AEM-1	*Penicillium citrinum*	MK396481	437
	AEM-3	*Rhizopus oryzae*	MK396483	613
	AEM-4	*Mucor circinelloides*	MK396484	648
	AEM-8	*Aspergillus sydowii*	MK396472	467
	AXM-1	*Aspergillus sydowii*	MK396475	546
	AMM-3	*Mucor indicus*	MK778442	565
*Hamei*	MHM-1	*Mucor circinelloides*	MK796043	601
	MHM-15	*Penicillium citrinum*	MK796042	469
*Chowan*	TCM-1	*Bjerkandera adusta*	MK396494	520
	TCM-4	*Mucor circinelloides*	MK778449	636
	TCM-7	*Rhizopus oryzae*	MK396491	637
	TCM-9	*Aspergillus sydowii*	MK796041	541
	TCM-12	*Penicillium chrysogenum*	MK778448	541
*Phut*	APM-1	*Aspergillus sydowii*	MK396473	577
	APM-3	*Mucor circinelloides*	MK396482	645
	APM-6	*Aspergillus versicolor*	MK396480	417
	APM-7	*Mucor indicus*	MK396498	627
	APM-12	*Rhizopus oryzae*	MK396490	621
	APM-15	*Aspergillus sydowii*	MK396474	574
*Dawdim*	MDM-1	*Mucor circinelloides*	MK396497	645
	MDM-10	*Bjerkandera adusta*	MK396493	569
	MDM-11	*Rhizopus microsporus*	MK396488	696
	MDM-14	*Mucor circinelloides*	MK396486	641
	MDM-16	*Bjerkandera adusta*	MK396499	680
	MDM-18	*Penicillium chrysogenum*	MK778443	554
*Khekhrii*	NKM-1	*Mucor circinelloides*	MK796045	490
	NKM-6	*Penicillium citrinum*	MK396479	519
	NKM-7	*Aspergillus flavus*	MK396470	519
	NKM-8	*Aspergillus niger*	MK396471	551
	NKM-10	*Penicillium oxalicum*	MK778444	581
	NKM-13	*Aspergillus niger*	MK396476	602
	NKM-15	*Cladosporium parahalotolerans*	MK796044	546

Frequency and density of fungal species in samples showed that *Aspergillus niger* was colonized with *khekhrii;* a species from the *Mucor circinelloides* complex was observed with a high dominance in samples, whereas *Trametes hirsuta* was less diversified and observed only in *thiat* samples (Table 3).

**TABLE 3:** Frequency, density, and diversity indices of filamentous molds observed in dry starters from North East India.

**Filamentous molds**	***Marcha***		***Thiat***		***Humao***		***Hamei***		***Chowan***		***Phut***		***Dawadim***		***Khekhari***	
	**%**															

	**Fr**	**RD**	**Fr**	**RD**	**Fr**	**RD**	**Fr**	**RD**	**Fr**	**RD**	**Fr**	**RD**	**Fr**	**RD**	**Fr**	**RD**
*Aspergillus niger*	0	0	0	0	0	0	0	0	0	0	0	0	0	0	25	0.25
*Aspergillus flavus*	16.6	0.16	0	0	0	0	0	0	0	0	0	0	0	0	12.5	0.12
*Aspergillus sydowii*	0	0	16.6	0.16	33.3	0.33	0	0	20	0.2	33.3	0.33	0	0	12.5	0.12
*Aspergillus versicolor*	0	0		0	0	0	0	0		0	16.6	0.16	0	0	0	0
*Penicillium chrysogenum*	16.6	0.16	16.6	0.16	0	0	0	0	20	0.2	0	0	16.6	0.16	0	0
*Penicillium citrinum*	0	0	0	0	16.6	0.16	50	0.5	0	0	0	0	0	0	12.5	0.12
*Penicillium oxalicum*	0	0	0	0	0	0	0	0	0	0	0	0	0	0	12.5	0.12
*Cladosporium parahalotolerans*	0	0	0	0	0	0	0	0	0	0	0	0	0	0	12.5	0.12
*Penicillium polonicum*	16.6	0.16	0	0	0	0	0	0	0	0	0	0	0	0	0	0
*Mucor circinelloides*	16.6	0.16	16.6	0.16	16.6	0.16	50	0.5	20	0.2	16.6	0.16	33.3	0.33	12.5	0.12
*Mucor indicus*	0	0		0	16.6	0.16	0	0	0	0	16.6	0.16	0	0	0	0
*Rhizopus oryzae*	0	0		0	16.6	0.16	0	0	20	0.2	16.6	0.16	0	0	0	0
*Rhizopus delemar*	0	0	16.6	0.16	0	0	0	0	0	0	0	0	0	0	0	0
*Rhizopus microsporus*	16.6	0.16667		0	0	0	0	0	0	0	0	0	16.6	0.16	0	0
*Trametes hirsuta*	0	0	16.6	0.16	0	0	0	0	0	0	0	0	0	0	0	0
*Bjerkandera adusta*	16.6	0.16667	16.6	0.16	0	0	0	0	20	0.2	0	0	33.3	0.33	0	0
**DIVERSITY INDICES**																
Species richness (R)	6		5		5		2		5		5		4		6	
Shannon’s diversity index (H)	1.74		1.6		1.56		0.69		1.6		1.56		1.32		1.46	
Species evenness (E)	0.97		1		0.96		1		1		0.96		0.95		0.82	

**Fr, Frequency of fungal species; RD, Relative density of fungal species in samples.**

Diversity indexes of filamentous molds of dry starters were characterized by species richness (R), Shannon’s diversity index (H), and species evenness (E) (Table 3). The Shannon diversity index *H* was recorded as the highest in *marcha* from Sikkim (*H*: 1.74) and the lowest in *hamei* from Manipur (*H*: 0.69). Species Evenness (E) values were 0.97 in *marcha* followed by *humao* from Assam and *phut* from Arunachal Pradesh. The Species Richness (R), values were recorded highest in *marcha* and *khekhrii* samples (Table 3).

## Discussion

Drinking of cereal-based mild to strong alcoholic beverages produced by traditionally prepared amylase and alcohol-producing starters has been a traditional food culture of the ethnic people from the North East states of India for centuries. Traditionally prepared dry starters have consortia of co-existed microbiota containing filamentous molds, yeasts, and bacteria and are crudely sub-cultured through a “back-slopping” process by traditional starter-makers (Hesseltine et al., 1988; Tamang and Sarkar, 1995; Tamang et al., 2007; Sha et al., 2018, 2019), for alcohol production by the Indian people. The pH of traditionally prepared dry starters from India were slightly acidic in nature, perhaps due to accumulation of metabolic organic acids (Ma et al., 2019). Moreover, low pH is favorable for the growth of mycelial fungi (Abubakar et al., 2013). Low content of moisture in starter cultures is due to the sun-drying process during the traditional method of preparation practiced by the ethnic people of India, which may increase the shelf life of the starter for a year or more at room temperature (Tsuyoshi et al., 2005; Tamang, 2010).

Some traditionally prepared starters from North East India have been microbiologically analyzed in earlier works and several species of yeasts (Tsuyoshi et al., 2005; Jeyaram et al., 2008, 2011; Sha et al., 2017, 2018, 2019) and bacteria (Tamang et al., 2007; Pradhan and Tamang, 2019) were reported. However, detailed taxonomical studies of filamentous molds isolated from traditionally prepared dry starters from North East India have not been reported yet, except for *marcha* (Tamang et al., 1988; Tamang and Sarkar, 1995; Sha et al., 2017, 2019), *thiat* (Sha et al., 2017, 2019), *amou, perok-kushi* (Das et al., 2017). Hence, we studied the taxonomy and diversity of filamentous fungi associated with traditionally prepared dry starter cultures from North East India viz., *marcha* from Sikkim, *thiat* from Meghalaya, *humao* from Assam, *hamei* from Manipur, *chowan* from Tripura, *phut* from Arunachal Pradesh, *dawdim* from Mizoram, and *khekhrii* from Nagaland based on morphological characters and molecular identifications. The average fungal population in traditionally prepared dry starters from North East India was 10^5^ cfu/g, which was in accordance with earlier reports on fungal populations in *marcha* of Sikkim, and the Darjeeling hills in India (Tamang et al., 1988; Tamang and Sarkar, 1995). No such data on fungal population in other starters of India are available except for *marcha*. In the present study, we first isolated and characterized 131 fungal isolates from 40 different starters from North East India based on macroscopic and microscopic characteristics and grouped them into 44 representative fungal strains. Morphological examination and identification of fungi are useful for identification up to the family or genus level (Alsohaili and Bani-Hasan, 2018). However, morphological-based identification is not adequate to identify the fungi up to species level (Lutzoni et al., 2004). The sequence-based identification tool is widely applied to confirm the exact identify of the fungal species (Romanelli et al., 2010; Xu, 2016).

We applied polymerase chain reactions (PCR) of the internal transcribed spacer (ITS) region of 44 strains of filamentous fungi isolated from starters from North East India using the primers ITS1 and ITS4 and grouped into three phyla represented by Ascomycota (48%), Mucoromycota (38%), and Basidiomycota (14%). A similar type of phylum distribution was also reported earlier in a *nuruk* dry starter from Korea (Carroll et al., 2017) and *daqu* from China (Shoubao et al., 2019). Seven genera with 16 species of filamentous fungi, isolated from Indian amylase and alcohol-producing starters, were identified based on the morphological and microscopic characteristics, and molecular identification which were represented by *Aspergillus flavus, A. niger*, *A. sydowii*, *A. versicolor*, *Bjerkandera adusta*, *Cladosporium parahalotolerans*, *Mucor circinelloides*, *M. indicus*, *Penicillium chrysogenum*, *P. citrinum*, *P. oxalicum*, *P. polonicum, Rhizopus delemar, R. microsporus, R. oryzae*, and *Trametes hirsuta*. Illustration of taxonomical keys based on morphological and molecular identification is more accurate and reliable in fungal taxonomy (Xing et al., 2018). Our earlier findings of *Rhizopus oryzae* and species from the *Mucor circinelloides* complex in traditionally prepared starters of North East India by PCR-DGGE method (Sha et al., 2018) supported the present study. Hesseltine and Kurtzman (1990) reported species from the *M. circinelloides* complex in *bubod* from the Philippines. Species from the *M. circinelloides* complex, *M. indicus, Rhizopus oryzae*, and *R. microsporus* were reported i*n benh men* from Vietnam (Dung et al., 2007; Thanh et al., 2008). In *marcha* and *khekhrii* we detected *Aspergillus flavus*, which was also reported in *mana*, an amylolytic starter from Nepal (Nikkuni et al., 1996).

*Aspergillus* belonging to order Eurotials is a phenotypically polythetic genus and is widely distributed in the environment (Tsang et al., 2018). Samson et al. (2014) proposed phylogenic identification of *Aspergillus* with ITS sequence data, and calmodulin as a secondary identification marker, according to the decision of the International Commission of *Penicillium* and *Aspergillus*^2^. Application of ITS with β-tubulin sequences for identification of *Aspergillus* species has also been reported by Zulkifli and Zakaria (2017). However, in this study we have applied both ITS sequence and morphological characteristics, such as the conidiophore with straight ending in a large vesicle from where primary and secondary sterigmata arise bearing conidia in chains, for identification of species of *Aspergillus*. *Aspergillus niger* and *A. flavus* cannot be distinguished only by their ITS sequences, the morphological characters are also essential in species identification (Zulkifli and Zakaria, 2017). We identified genus *Aspergillus* with four species in dry starter samples from India which included *A. niger*, *A. flavus*, *A. sydowii*, and *A. versicolor*. Among *Aspergillus A. flavus, A. niger* and *A. sydowii* were most prevalent in food samples due to their sporulating ability in the environment (Adekoya et al., 2017). *Aspergillus* is a dominant fungal genus in *daqu* from China (Ji et al., 2018), and may contribute to the saccharification process (Wang et al., 2019). We detected two strains of *Aspergillus flavus* in a *marcha* sample from Sikkim (*Aspergillus flavus* SMM-1) and in a *khekhrii* sample from Nagaland (*A. flavus* NKM-7). Though the distribution percentage was only 5%, the presence of *A. flavus* in samples of *marcha* and *khekhrii* is alarming. *A. flavus* is a saprotrophic with cosmopolitan distribution (Ramírez-Camejo et al., 2012), which produces aflatoxin (Saori and Keller, 2011; Priyanka et al., 2012; Mudili et al., 2014). Probable sources of *A. flavus* in starters may be from contaminated rice grains (Lai et al., 2015) since rice is the main base substrates for the preparation of starters for the production of alcohol. Moreover starter-makers commonly use low-quality, old-stocked and discarded rice grains for preparation of starters. However due to co-existence of other species of filamentous molds, yeasts and lactic acid bacteria in traditionally prepared starters may antagonize against *A. flavus* in *marcha* and *khekhrii*, which may reduce aflatoxin production in the sample (Karlovsky et al., 2016; Adebo et al., 2019). Lactic acid bacteria isolated from *marcha* showed an antagonistic property (Tamang et al., 2007), similarly, some bacteria have antifungal activity against aflatoxin-producing *A. flavus* (Shakeel et al., 2018). *Rhizopus* spp. from *tempeh*, a fermented soybean food from Indonesia, were reported for detoxification of alfatoxins (Nakazato et al., 1990). *A. sydowii* present in samples *humao*, *phut* and *chowan*, is an industrially important filamentous mold, which produces monosaccharides and indole alkaloids (Zhou et al., 2018). None of the amylolytic starters of North East India showed the presence of *A. versicolor* except in *phut* samples from Arunachal Pradesh. *A. versicolor* is a slow-growing filamentous fungus commonly found in/on damp indoor environments (Samson et al., 2004), foods, and feeds (Jurjevic et al., 2012), and produces toxic metabolites (Piontek et al., 2016). Contamination of *A. versicolor* in *phut* samples might be from the damp room where preparation of *phut* is often practiced by starter-producers in Arunachal Pradesh.

*Mucor circinelloides* was found to be the most dominant fungus in dry starter cultures from North East India. *M. circinelloides* has a sub-globose sporangiospore with a sympodial branching pattern. Using the ITS sequencing tool, it is difficult to distinguish among the different species of the *Mucor circinelloides* complex (MCC) which include *M. circinelloides, M. griseocyanus, M. janssenii, M. lusitanicus, M. ramosissimus, M. variicolumellatus*, and *M. velutinosus* (Wagner et al., 2019). We therefore used species from the *Mucor circinelloides* complex. *Mucor circinelloides* contributes in saccharification and liquefaction of cereal during fermentation of *kodo ko jaanr*, an alcoholic product of Sikkim fermented by starter *marcha* (Thapa and Tamang, 2004; Tamang and Thapa, 2006). *M. circinelloides* is an oleaginous fungus (Qiao et al., 2018) which produces lipids (Wei et al., 2013), cellulose degrading enzymes (Huang et al., 2014), and has several functional properties including antioxidants (Hameed et al., 2017). Phylum Mucoromycota does not produce mycotoxins, however, some species that belong to this *M. circincelloides* forma *circinelloides* group has been described to be putatively responsible for human illnesses after consumption of mold-contaminated yogurt (Lee et al., 2014) although its involvement was not clearly proven. *M. circinelloides* was also reported earlier in *marcha* samples (Tamang et al., 1988; Tamang and Sarkar, 1995). *M. indicus*, isolated from *humao* from Assam and *phut* from Arunachal Pradesh, is a dimorphic and ethanolic fungus which is able to produce ethanol from glucose, mannose, fructose and galactose (Karimi and Zamani, 2013) and oil, protein, and glucosamine (Sharifyazd and Karimi, 2017).

Phylogenetic and phylogenomic approaches show that genus *Rhizopus* has three major clades viz. *R. microsporus with* its sister taxon *R. stolonifer*, *R. arrhizus*, and *R. delemar* (Gryganskyi et al., 2018). *Rhizopus oryzae*, commonly inhabits soils, animal excrement, and rotting vegetables (Ghosh and Ray, 2011), and is very similar to *Rhizopus stolonifer*, except for its smaller sporangia with air-dispersed sporangiospores (Pitt and Hocking, 2009). *R. oryzae* and *R. microsporus* are detected in *yao qu* from China and *banh men* from Vietnam, which are strong amylase producers (Dung et al., 2007; Thanh et al., 2008; Lv et al., 2012b). *R. oryzae* is considered as a GRAS filamentous fungus (Londoño-Hernández et al., 2017), which is commonly used for production of some Asian fermented foods (Tamang et al., 2016). *Rhizopus microsporus* is the major fungus in *tempe*, a fermented soybean food from Indonesia (Hartanti et al., 2015). *R. delemar* was found in the *thiat* sample only, which naturally accumulates fumaric acid with a fruity taste (Odoni et al., 2017), and it probably imparts taste and flavor in *kiad*, an alcoholic product fermented by the starter *thiat. R. delemar* has also been reported in *xajpitha*, starter from Assam in India (Bora et al., 2016). Presence of *Rhizopus* spp. in starters from North East India may contribute functionalities in end products during acholic fermentation.

*Penicillium chrysogenum* was found in only four types of starters viz. *marcha* (Sikkim), *thiat* (Meghalaya), *chowan* (Tripura), and *dowdim* (Mizoram). The probable entry of *P. chrysogenum* during traditional preparation may be from damp and moist rooms where preparation for such starters is usually done, since *P. chrysogenum* is also found in damp buildings (Andersen et al., 2011). Due to the ability of *P. chrysogenum* to produce antibiotics, mostly penicillin (Bajaj et al., 2014), its presence in starters may have an antagonist property in the end product. *P. citrinum* was recovered in samples of *humao, hamei* and *khekhrii*, probably from indoor environments (Samson et al., 2004). *P. oxalicum* was found in samples of *khekhrii* (Nagaland) and *P. polonicum* in *marcha* samples. *P. oxalicum* produces various enzymes and natural products (Li et al., 2016). *P. polonicum* has also been reported in fermented black table olives (Bavaro et al., 2017).

It is interesting to note that we detected Basidiomycetous fungi represented by *Bjerkandera adusta* in samples of *marcha, thiat, dawdim*, and *chowan*, and also *Trametes hirsuta* in *thiat* samples. *Bjerkandera adusta* and *Trametes hirsuta* are wood decaying white-rot fungi (Rosales et al., 2005; Horisawa et al., 2019). *B. adusta* grows on a natural cellulosic substrate, imparts a refreshing aroma (Zhang et al., 2015), contributes to saccharification (Quiroz-Castañeda et al., 2009), and produces ethanol (Horisawa et al., 2019). *Trametes hirsuta* is lignin-degrading fungus due its ability to synthesize laccase (Cilerdzic et al., 2011). Traditional methods of preparation of these amylolytic starter cultures require locally grown wild herbs and spices used as ingredients by local starter-makers (Anupma et al., 2018). We assume that during collection of wild herbs from forest grounds, people might have collected whole wild plants *in situ*, where wood-rooting fungi have been reported in forests of North East India (Chuzho et al., 2017). There is no practice of filtering and cleaning of collected wild plants during starter preparation, hence chances for contamination of these basidiomycetous fungi may be possible during preparation. *B. adusta* and *T. hirsuta* were not reported earlier in any starter culture or in any fermented food.

*Cladosporium parahalotolerance* was found only in samples of *khekhrii*. *C. parahalotolerance* mostly occurred in plant debris, foods, and indoors (Bensch et al., 2012). Source of *Cladosporium* in *khekhrii* might be from wild herbs used as ingredients during traditional preparation of *khekhrii* in Nagaland. Species of *Bjerkandera, Trametes*, and *Cladosporium* have not been reported in any fermented foods elsewhere.

Diversity indexes determine the phylogenetic relations within different fungal species in a community (Fernandes et al., 2015). We calculated diversity indexes of fungal community present in starters of North East India by Shannon’s diversity index (H), species evenness (E), and species richness (R). Shannon diversity index *H* for evaluating fungal diversity was recorded highest in *marcha* samples collected from Sikkim (H: 1.74) and lowest in *hamei* samples of Manipur (H: 0.69) indicating higher fungal diversity in *marcha* samples of Sikkim as compared to starters of other states. The diversity index, which considers both the number of species as well as relative abundance of each species for evaluating diversity (Lucas et al., 2017), showed the highest value for *marcha* of Sikkim. Species richness is the number of different species represented in an ecological community, where it reflects the abundances of species or their distributions (Unterseher et al., 2008). Species Richness (R) values in samples of *marcha* and *khekhrii* were recorded as the highest showing more diversity in species level of filamentous molds. Species evenness refers to how equal the community is numerically, ranging from 0 to 1 (Savary et al., 2018) signifying that the value 1.0 in *thiat, hamei*, and *chowan* have a complete evenness in comparison to other starters. Hence diversity index of filamentous fungal community present in dry starters of North East India showed high diversity within the community. It was observed that there was variation in fungal species distribution in each type of amylolytic starters in North East India which determines the quality of the acholic product, preferred by the local consumers. This might be due to varied geographical regions, environmental conditions, and different plant species used in the preparation methods of amylolytic starters. It therefore shows that fungal diversity, present in amylase and alcohol-producing starters, traditionally prepared by ethnic Indian people using their indigenous knowledge of “back-slopping,” are morphologically, ecologically, and phylogenetically diverse. Our findings on fungal diversity in amylolytic starters from North East India may supplement the microbial diversity in eco-systems of North East India, which is one of the biodiversity hot spots of the world.

## Conclusion

Traditionally prepared amylolytic starters are consortia of filamentous fungi, yeasts, and bacteria which are traditionally sub-cultured and preserved using traditional methods of “back-slopping” by the ethnic people of North East India for production of alcoholic beverages. Yeasts and bacteria present in these starters have already been reported in earlier studies. However, no information on fungal communities and their diversity in Indian amylolytic starters is available. We therefore identified the filamentous molds isolated from *marcha, thiat, humao*, *hamei*, *chowan*, *phut*, *dawdim*, and *khekhrii* based on morphological and sequence-based identifications. We identified seven genera with 16 species represented by *Aspergillus flavus, Aspergillus niger*, *Aspergillus sydowii*, *Aspergillus versicolor*, *Bjerkandera adusta*, *Cladosporium parahalotolerans*, *Mucor circinelloides*, *Mucor indicus*, *Penicillium chrysogenum*, *Penicillium citrinum*, *Penicillium oxalicum*, *Penicillium polonicum, Rhizopus delemar, Rhizopus microsporus, Rhizopus oryzae*, and *Trametes hirsuta*. Fungal species present in these traditionally prepared dry starters are morphologically, ecologically, and phylogenetically diverse and showed high diversity within the community.

## Data Availability Statement

The sequences of the internal transcribed spacers (ITS) region obtained in this study were deposited at the GenBank-NCBI database 6S rRNA sequencing were deposited at GenBank-NCBI numbers: MK396469-MK396484, MK396486-MK396500, MK778442-MK778449, MK796041-MK796045.

## Author Contributions

AA performed the experiments. JT supervised the experiments and finalized the manuscript.

## Conflict of Interest

The authors declare that the research was conducted in the absence of any commercial or financial relationships that could be construed as a potential conflict of interest.

## Footnotes

1 http://www.ncbi.nlm.nih.gov/Blast.cgi

2 www.aspergilluspenicillium.org
